# Handling ligands with *Coot*

**DOI:** 10.1107/S0907444912000200

**Published:** 2012-03-16

**Authors:** Judit É. Debreczeni, Paul Emsley

**Affiliations:** a Structure and Biophysics, DS, AstraZeneca, Alderley Park SK10 4TG, England; b Department of Biochemistry, University of Oxford, South Parks Road, Oxford OX1 3QU, England

**Keywords:** *Coot*, ligands

## Abstract

*Coot* is a molecular-graphics application primarily aimed to assist in model building and validation of biological macromolecules. Recently, tools have been added to work with small molecules. The newly incorporated tools for the manipulation and validation of ligands include interaction with *PRODRG*, subgraph isomorphism-based tools, representation of ligand chemistry, ligand fitting and analysis, and are described here.

## Abstract 2

*Coot* is a molecular-graphics program designed to assist in the building of protein and other macromolecular models. The current state of ligand tools is presented.

## 1. Introduction

The use of protein crystallography for the optimization of potential drug-candidate molecules has been well established (Congreve *et al.*, 2005 ▶). In recent years, the use of high-throughput (HT) crystallographic techniques has enabled the delivery of structural data in a timely fashion and many drug-discovery programmes have progressed in the light of information from target-ligand complex structures (Tickle *et al.*, 2004 ▶; Williams *et al.*, 2005 ▶). The combination of HT methods and a fragment-based approach has extended the utility of structural biology as a screening technique for the identification of novel small-molecule binders of medically relevant target proteins.

Although the pharmaceutical industry has used ligand structures successfully, it has been shown that the quality of small molecules in protein–ligand complexes varies widely in the worldwide Protein Data Bank (wwPDB; Berman *et al.*, 2003 ▶) and therefore the wwPDB cannot always be considered to be a reliable repository of structural information pertaining to small molecules (Cooper *et al.*, 2011 ▶).

The determination of protein–ligand complex structures has mostly been automated (see, for example, Mooij *et al.*, 2006 ▶) but still requires user intervention at various stages if the active site has substantial flexibility.

Here, we describe the new tools in *Coot* that are designed to assist with ligand model building. These tools illustrate chemistry, handle fragments and interact with programs of the *CCP*4 suite (Winn *et al.*, 2011 ▶). The tools presented here assist with the generation, validation and manipulation of protein–ligand complex structures with a view to increasing ‘crystallo­graphic intelligence’ and automation.

## 2. Restraints

The basics and necessity of stereochemical restraints have been laid out by Evans (2007 ▶) and Kleywegt (2007 ▶). Briefly, the restraints that are typically used describe ideal values and estimated standard deviations for bond lengths, bond angles, torsion angles, planar groups and chiral volumes. The generation of high-quality restraints for heterocompounds remains a substantial stumbling block.

Restraints for all compounds in the wwPDB (at the time of release) are available in the new version of the dictionary supplied with the program *REFMAC* (Vagin *et al.*, 2004 ▶) distributed by *CCP*4 in v.6.2. An alternative source is the web service HIC-Up (Kleywegt & Jones, 1998 ▶). The standard dictionary types produced by HIC-Up are those for the programs *O* (Jones *et al.*, 1991 ▶) and *X-PLOR* (Brünger, 1992 ▶).

## 3. Generating ligand descriptions with *Coot*

The task of ligand fitting to a protein structure often starts with the generation of a representation of the ligand. The three-dimensional coordinates of a ligand conformer and the dictionary can be generated by a number of means depending on the starting point [typically SMILES or an MDL Molfile (a simple two-dimensional molecular description specifying atom elements and bond orders)]. Software to convert from these starting points (and, in some cases, further steps in the ligand-fitting process) include the programs *CORINA* (Gasteiger *et al.*, 1990 ▶), *LIBCHECK* (Vagin *et al.*, 2004 ▶), *PRODRG* (Schüttelkopf & van Aalten, 2004 ▶), *phenix.elbow* (Moriarty *et al.*, 2009 ▶) and *AFITT* (Open Eye Scientific Software; Wlodek *et al.*, 2006 ▶). These programs not only produce a three-dimensional model for the ligand but also, and just as importantly, a restraints dictionary.

An alternative starting point is a copy of the three-dimensional model coordinates of the ligand. This starting point involves the perception of chemistry and is not handled by current *Coot* tools.

### 3.1. Two-dimensional ligand sketcher

*Coot* has a two-dimensional ligand sketcher, along the lines of the *MarvinSketch* software from ChemAxon or the JME molecular-editor application (http://www.molinspiration.com/jme/), for the free drawing of chemical diagrams. The editor has a built-in knowledge of chirality, valence, charge and hydrogen assignment. It allows the import and export of structures in SMILES and the MDL Molfile format.

*Coot* also provides a programmatic interface (both Scheme and Python) to receive coordinate and dictionary ‘objects’: these are convenient for program-to-program communication (*i.e.* obviating the need to read and write molecule files to the file system).

### 3.2. Interface to *PRODRG*

The *CCP*4 software suite now includes *PRODRG* (Schüttelkopf & van Aalten, 2004 ▶) as a command-line-driven program. The two-dimensional ligand sketcher in *Coot* interacts with *PRODRG* on behalf of the user, providing the MDL Molfile as input to *PRODRG* and reading and displaying the output [namely a PDB file (Bernstein *et al.*, 1977 ▶) containing three-dimensional coordinates and a CIF restraints dictionary]. It should be noted that *PRODRG* does not handle ligands with metal atoms.

*Coot* uses *LIBCHECK* to generate molecules when starting from a SMILES string.

## 4. Common subgraph isomorphism-based tools

Chemical structures can straightforwardly be represented as mathematical graphs, with the graph edges and vertices representing the molecule’s bonds and atoms. Using an improved backtracking algorithm in the common subgraph isomorphism (CSI) search (Krissinel & Henrick, 2004 ▶) has considerably increased search speed over more traditional methods.

This technique has enabled a number of tools that are useful in ligand comparison and manipulation.

### 4.1. SBase searching

SBase is a database containing descriptions of practically all residues and small-molecule component types found in the PDB as of 2007. SBase, distributed by the PDBe, has an equivalent information content as the wwPDB’s Chemical Component Dictionary. However, SBase has the advantage of being rapidly accessible and has a C++ API available in the mmdb library from the *CCP*4 suite of programs (Krissinel *et al.*, 2004 ▶) that also provides access to CSI. This API is exploited by *Coot*.

Using the two-dimensional ligand sketcher (or by importing an MDL Molfile) one can generate a chemical diagram that can be used to search SBase given a user-defined similarity fraction (at least a given percentage of the atoms in the search fragment have corresponding atoms in the database fragment). Compounds returned by the search can be imported into *Coot* as three-dimensional models together with their restraints description.

A search against bespoke or internal databases is not possible at the time of writing, but should be available in the future.

### 4.2. Least-squares ligand overlay

*Coot* uses the CSI search from the mmdb library to identify the core fragment of a reference molecule that matches that of a ‘moving’ ligand. Provided that there are more than two such atom pairs, *Coot* uses the atom-pair list to provide a rigid-body rotation–translation matrix that transforms the ‘moving’ ligand.

### 4.3. Atom-name mapping

When comparing similar ligands in a binding pocket, it is convenient that structurally equivalent atoms are named identically (*e.g.* the label ‘C2’ refers to ‘the same’ atom in the ligands that are being compared). However, certain ligand description-generating programs (*e.g. PRODRG*) arbitrarily rename atoms and do not maintain a consistent naming scheme with reference ligands.

Like the ligand-overlay tool, the atom-name-mapping tool matches the atoms of a ‘working’ molecule to those of a reference molecule. The atom names of the ‘working’ ligand are then changed to those that correspond to the reference ligand. To maintain the uniqueness of atom names, new atom names are substituted in the ‘working’ molecule if the non-core atom names of the ‘working’ molecule are the same as the core atoms of the reference molecule.

### 4.4. Torsion matching

Again using the CSI match, *Coot* identifies matching atoms between a ‘working’ and a reference ligand. When complete restraints descriptions of both ligands are available, the torsion angles can be compared. The description of each rotatable torsion angle (*i.e.* those that are not marked as ‘const’ in the CIF dictionary file) in the reference ligand is examined to see if it has a counterpart in the ‘working’ ligand torsion-angle description by way of atom-name matching. If there is such a match, by using internal coordinate manipulation the matching torsion of the ‘working’ ligand is set to that of the reference ligand. It should be noted that currently not all molecules work well with torsion matching (*e.g.* pyranoses) and this is under investigation.

## 5. Ligand chemistry representation

Chemical compounds require more detailed description than that used to represent the protein and are more suitably described with bond orders represented, whereas for the protein this is typically not required or may even be a hindrance (since the bond orders of protein side chains are well understood). To this end, *Coot* uses the bond-order description in the dictionary when displaying ligands (‘single’, ‘double’, ‘triple’, ‘metal’, ‘deloc’ and ‘aromatic’ are the known types of bonds). If aromatic bonds are described as ‘aromatic’ instead of alternating single and double bonds then the aromatic ring-detection system is activated and the bond system is displayed with a ring.

While H atoms are important in the interaction of a ligand with its environment, displaying them in a molecular-graphics system in the same manner as other atoms can make the view confusing and crowded. This problem has been recognized by the authors of the program *King* (Chen *et al.*, 2009 ▶), who found that the best representation for H atoms was a single-colour thin grey line to the H atom. *Coot* follows this representation style and applies it in a similar manner to the ball-and-stick representation.

**Figure 1:**
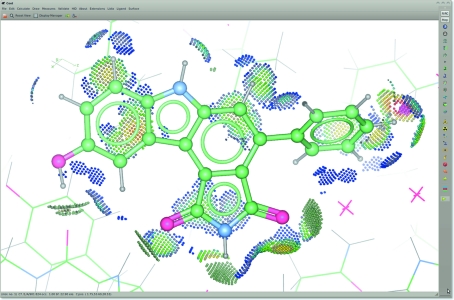
Screenshot of *Coot* demonstrating the representation of different chemical bond types and H atoms with smaller atom radii and monochrome bonds. Also shown are environment distances and isolated interaction dots (green, hydrogen bonds; blue, van der Waals contacts; red, close contacts; pink, clashes; carbazole-derivative ligand 824 in PDB entry 1x8b; Squire *et al.*, 2005 ▶).

These concepts are demonstrated in Fig. 1 ▶.

## 6. Restraints editor

*Coot* provides a tabular representation of the monomer restraint information, which makes it easier to review and manipulate restraints than by editing the CIF file in a text editor. The modified restraints can be applied to the residues manipulated in *Coot* and also written to a CIF file.

## 7. Ligand fitting

The core ligand-fitting algorithm of *Coot* has been described previously (Emsley & Cowtan, 2004 ▶). There are a number of ligand-fitting scenarios that *Coot* handles (Table 1 ▶). After conformer generation, the residual density map is searched for clusters of density grid points that might contain a ligand. These clusters are compared with the conformer shape *via* principal component analysis and the best conformers are accepted if they pass certain (user-definable) filters for density fit.

Currently, the interactions arising from the binding mode are not part of the scoring system in *Coot*’s ligand fitting. However, these metrics have been used to enhance the ligand fitting of other systems (Mooij *et al.*, 2006 ▶).

### 7.1. Conformer generation

The conformer of the ligand in the crystal structure may not match the conformation of the ligand generated by the aforementioned tools. Therefore, *Coot* generates conformers of the ligand, each of which is used to search the electron density. The conformer-sampling algorithm is simple-minded owing in part to the unsophisticated torsion probability distribution (derived from the restraints dictionary). However, the conformer internal energy is usually of little consequence compared with the fit to the electron density.

The torsion descriptions of the ligand are used to generate synthetic probability distributions. Each torsion angle is handled independently (torsions marked as ‘const’ are not varied). A value for the torsion of each torsionable bond is generated by random sampling from the probability distribution. Since the torsions are handled independently, this may lead to a high-energy conformation. Thus, as a final step, each conformation of the ligand undergoes energy minimization. To increase the probability of lower energy conformers, it is recommended that H atoms are used at this stage.

There is no special consideration made for ring torsions – random variation of the torsions of ring systems could well result in breaking of a ring bond – thus, for molecules with ring torsions the subsequent energy minimization (using *Coot*’s built-in gradient minimizer) is vital to create conformers that are chemically meaningful.

### 7.2. NCS ligands

*Coot* can take the noncrystallographic symmetry of protein chains into account when fitting ligands. When a ligand is placed into the active site of a single protein chain, the NCS relations can be used to generate coordinates for ligands in the active sites of the NCS-related chains.

### 7.3. Jiggle fit

Small and simple (*i.e.* containing only a few torsionable bonds) common solvent and cryo molecules (*e.g.* acetate, ethylene glycol and glycerol) can be placed and fitted in the electron density by a mouse click using the ‘Add other solvent molecules’ functionality. In such cases, instead of the more exhaustive ligand-fitting mechanism, jiggle fit is used to optimize the ligand’s fit to the density. The starting position of a low-energy conformer is transformed by applying a set of random rotations and small translations. The resulting positions are then submitted to rigid-body refinement and scored based on density fit. Finally, the best pose is real-space refined.

### 7.4. *JLigand* interface

If a bespoke link between a chemical compound and a protein residue needs to be made, the tool *JLigand* (Lebedev *et al.*, 2012 ▶) from the *CCP*4 suite can be invoked by clicking on the atoms to be linked.

After manipulation in *JLigand*, the resulting link information is automatically transferred between *JLigand* and *Coot*. Additionally, a link description can be incorporated in the coordinates file header in the form of a LINK record.

## 8. Ligand analysis

The atom-selection system of *Probe* can be used to isolate the ligand and provide a ‘*MolProbity* dots’ (Word, Lovell, LaBean *et al.*, 1999 ▶; Word, Lovell, Richardson *et al.*, 1999 ▶) representation of the interactions of the ligand with its environment. Such a representation highlights hydrogen bonds, van der Waals interactions and clashes (Fig. 1 ▶).

**Figure 2:**
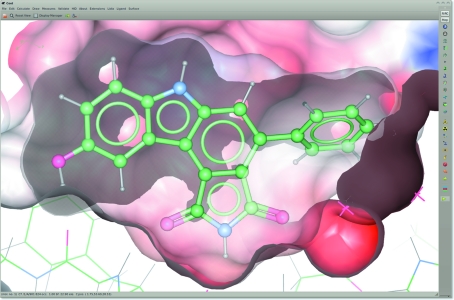
Surface representations in *Coot*. The protein electrostatic surface clipped to show the ligand-binding pocket of 824 in 1x8b. The ligand surface is transparent.

Protein–ligand shape and electrostatic complementarity can be examined using surface representations. *Coot* can map partial charges, read from the ligand dictionary, onto the surface representation. *Coot* also provides easy access to clipped surfaces that only cover the area of interaction, *e.g.* the protein’s surface clipped around the ligand to show the ligand-binding pocket. *Coot* also has an implementation of screendoor transparency for fast drawing of transparent surfaces (Fig. 2 ▶).

*Coot* contains a partial implementation of the two-dimensional layout system described by Clark *et al.* (2006 ▶) and Clark & Labute (2007 ▶).

## 9. Ligand validation

There are several validation methods for small-molecule substructures of protein–ligand complexes. One way to validate the geometry of ligand molecules is to compare the actual structure with its geometry description, *i.e.* restraints used in refinement. *Coot* calculates *Z* scores for all geometry features and represents the most disagreeing geometry features on a residue-by-residue basis in a chart form for all residues in the molecule (Validate → Geometry analysis…). This allows the user to quickly navigate to problematic areas of the structure; however, it cannot be used to detect issues in geometry parametrization.

If the restraint set used in refinement contains incorrect target values or inadequately set estimated standard deviation values, the resulting geometry might be distorted; hence, parametrization-independent validation is preferable for unprecedented residues where the restraint set is questionable. Parametrization-independent methods of small-molecule validation may involve comparison of the molecule in question to those found in previously solved structures or to calculated models, *e.g.* quantum-chemical calculations. While quantum-chemical calculations can be considered to be reliable in terms of the resulting geometry, they reflect an *in vacuo* state of the ligand which may not correspond to that found in the protein complex, which in turn could be flagged as erroneous.

Previously solved and deposited ligand structures provide another basis of comparison, as facilitated by, for example, the *ValLigURL* web service (Kleywegt & Harris, 2007 ▶). However, novel small molecules do not have any or enough representatives in the ligand set in the PDB, so this avenue is rather restricted to common ligands such as cofactors *etc*.

Validation of individual structural motifs against similar fragments of small-molecule crystal structures is an alternative way to assess ligand geometry. The program *Mogul* (Bruno *et al.*, 2004 ▶) from the Cambridge Crystallographic Data Centre (CCDC) is one of the knowledge bases that offer a quick and computationally inexpensive access to bond-length, bond-angle and torsion-angle values, as well as ring conformations. It also provides geometry distributions in a histogram form along with quality indicators (*Z* scores for bonds and angles and minimal distance values for torsions). Additions to the scripting interface in *Coot* allow the user to run *Mogul* in batch mode, to visualize the quality of the structural elements in the context of the electron density and surrounding residues in the structure and to navigate to the most disagreeable part of the residue of interest. Subsequently, the restraint set can be updated using the target values and standard deviations suggested by *Mogul*. In cases where the target value is close to ideal but the resulting geometry does not reflect this, the standard deviation is decreased, *i.e.* the restraint for that geometric element is tightened. One drawback of this method is that it relies on *Mogul*’s automatic chemistry perception; in other words, if the initial geometry is very far from ideal the detected atom types and bond orders might be incorrect. This can be overcome by using explicit H atoms on the residue to be validated.

## 10. Future developments

The set of ligand-handling tools currently implemented in *Coot* enable model building, refinement and analysis of organic small molecules. However, there are a number of developments planned to further enhance functionality and user experience.

The available ligand-representation styles are set to be extended with common styles found in other visualization software, *e.g.* the possibility of hiding nonpolar H atoms.

The current two-dimensional layout algorithm (for both the actual compound and its residue environment) can in certain cases fail to generate a clear and comprehensible depiction. The use of the RDKit library (http://www.rdkit.org) is being considered as a potential solution.

In the current implementation, SBase searches are restricted to the set of molecules published by the wwPDB. This will be extended to handle in-house or custom databases.

The CREDO database (Schreyer & Blundell, 2009 ▶) provides annotated information to describe protein–compound interactions and can be queried *via* the CREDO API. An interface will be developed for automated search and retrieval.

## Figures and Tables

**Table 1:** In the ‘known cocktail’ scenario, each ligand type is searched against each density cluster (and optionally involving conformer searching) At each of the residual density clusters, the best-fitting conformer of each of the ligand types is kept and presented to the user. *Coot* does not address the issue of unknown ligand types. The scenario of unknown ligand types is handled by the program *phenix.ligandfit* (Terwilliger *et al.*, 2007 ▶), which tests density blobs against a dictionary of 200 common heterocompounds.

	Ligand type known	Known cocktail	Ligand type unknown
Ligand position known	Yes	Yes	No
Ligand position unknown	Yes	Yes	No
